# Association of the epidermal growth factor gene +61A>G polymorphism with hepatocellular carcinoma in an Iranian population 

**Published:** 2017

**Authors:** Mehdi Gholizadeh, Ayyoob Khosravi, Pedram Torabian, Naghmeh Gholipoor, Nader Mansour Samaei

**Affiliations:** 1 *Depaetment of Human Genetics, Faculty of Advanced Medical Technologies, Golestan University of Medical Sciences, Gorgan, Iran *; 2 *Student Research Committee, Golestan University of Medical Sciences, Gorgan, Iran*; 3 *Department of Molecular Medicine, Faculty of Advanced Medical Technologies, Golestan University of Medical Sciences, Gorgan, Iran*; 4 *National Institute of Genetic Engineering and Biotechnology, Department of Molecular Genetics, Tehran, Iran*

**Keywords:** Epidermal growth factor, EGF +61A>G polymorphism, Hepatocellular carcinoma

## Abstract

**Aim:**

The aim of this study was to address the association of the EGF gene +61A/G polymorphisms and HCC susceptibility in an Iranian population.

**Background:**

The association of epidermal growth factor (EGF) gene +61A/G polymorphism (rs4444903) and hepatocellular carcinoma (HCC) has been investigated in several populations. However, the findings are controversial.

**Methods:**

A total of 40 unrelated HCC patients and 106 healthy individuals were enrolled in this study. Genomic DNA of HCC patients was extracted from formalin-fixed, paraffin-embedded samples using CinnaPure DNA kit according to manufacturer’s instructions. Genomic DNA of healthy individuals, also, was extracted from peripheral blood cells using the boiling method. The rs4444903 (A/G) polymorphism was genotyped using the polymerase chain reaction (PCR)-restriction fragment length polymorphism (RFLP) method.

**Results:**

Significant association was found for the EGF +61A allele and HCC risk [OR = 1.72, 95% CI (1.02 - 2.90), P value = 0.04]. Also, significant association was observed for the EGF +61A/G genotypes and HCC risk under codominant and dominant models by SNPStats software analysis.

**Conclusion:**

Our findings suggest that the EGF gene +61A/G polymorphism (rs4444903) might be a risk factor for susceptibility to HCC in Iranian population. However, further studies using more samples are needed.

## Introduction

 Hepatocellular carcinoma (HCC) is the third leading cause of cancer-related deaths and the fifth common solid tumor worldwide (1). The incidence and distribution of HCC vary among different countries (2), with the highest incidence reported in Asian and African population (3). The incidence of HCC has been estimated less than 10 cases per 100,000 population in North America and Western Europe as well as Iran؛ In parts of Africa and Asia it reaches 50 – 150 cases per 100,000 population (3). A variety of environmental and genetic factors are involved in the pathogenesis of HCC. Many environmental factors including chronic hepatitis B virus (HBV) and hepatitis C virus (HCV) infections, intense alcohol addiction, diabetes, obesity and tobacco use have been shown to be involved in HCC development (4-7). Genetic factors also play an important role in HCC pathogenesis (2). The identification of genetic factors influencing the risk of developing HCC can help to improve prevention and treatment strategies. The presence of specific single nucleotide polymorphisms (SNPs) in human genes has been associated with susceptibility to carcinogenesis (8). The associations between SNPs and the risk of HCC have been reported in several studies (9). 

Epidermal growth factor (EGF) plays a critical role in cell proliferation, differentiation, and tumorigenesis of epithelial tissues (10). EGF is overexpressed during development of HCC as a mitogen for hepatocytes (10-12). Mounting evidence supports a role for EGF in malignant transformation and tumor progression (13-15). Genetic variants in the EGF gene play critical roles in carcinogenesis. The EGF 61A>G polymorphism (rs4444903) is a commonly functional SNP in the 5’ untranslated region of the EGF gene that results in higher EGF levels in individuals with EGF genotype G/G in comparison to the A/A genotype and affects individual susceptibility to various carcinomas (16-19). Previous studies showed that EGF rs4444903 could result in increased risk of tumorigenesis in HCC. (20-22). However, some studies have indicated that the rs4444903 polymorphism has no significant association with HCC (20). The aim of this study was to address the association between EGF +61A>G polymorphism and HCC in an Iranian population. 

## Methods

**Study Subjects**

In this case-control study, 146 Iranian subjects including 40 unrelated HCC patients and 106 healthy individuals were investigated for genetic variations in the EGF gene +61A/G polymorphisms. The study was approved by the Ethics Committee of Golestan University of Medical Sciences (No: 14791793061990) and written informed consent was obtained from all patients and healthy individuals. The HCC patients were recruited from the seven Iranian hospitals (Mazandaran, Tehran, Shiraz, Esfehan, Sistan and Baluchestan and Gorgan). All patients were positive for HCV and had a history of choronic liver diseases. Data abstracted from medical records of patients. They did not have any other types of liver diseases such as alcoholic liver diseases, autoimmune liver diseases, or metabolic liver diseases. HCC patients were diagnosed by expert physicians based on pathologic and histopathologic criteria (23). The control group was matched with HCC patients for age, sex and ethnical origin. 

**DNA extraction**

Genomic DNA of HCC patients was extracted from formalin-fixed, paraffin-embedded (FFPE) tumor samples using CinnaPure DNA kit (Sinnaclone, Iran) according to manufacturer’s instructions. Genomic DNA of healthy individuals, also, was extracted from peripheral blood cells using the boiling method. DNA concentration was determined by Picodrop UV/Vis Spectrophotometer (Picodrop Ltd, UK).

**Genotyping**

The EGF +61A>G (rs4444903) polymorphism was genotyped using the polymerase chain reaction (PCR)-restriction fragment length polymorphism (RFLP) method, as previously described (24). Primers used were as follows: 5'-TGTCACTAAAGGAAAGGAGGT-3' (Forward) and 5'-TTCACAGAGTTTAACAGCCC-3' (Reverse) (18). PCR was performed using the following conditions: 95° C for 5 min followed by 30 cycles of 95° C for 30 s, 60° C for 30 s, 72° C for 40 s and a final extension step at 72° C for 5 min. 

**Figure 1:**
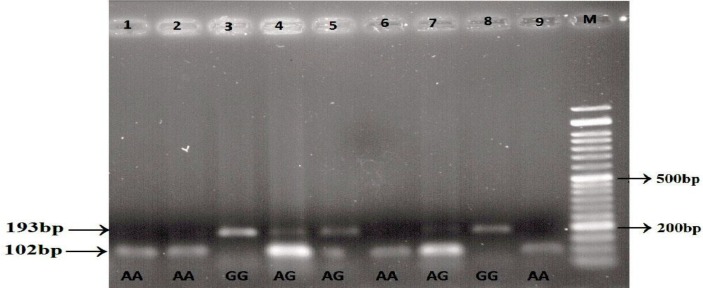
The genotyping process. The RFLP products of the EGF (rs444903) amplicons with AluI, is shown on 3% agarose gel. M represents the DNA size marker (50 bp

Subsequently, 1µg of the amplified product was digested with the AluI restriction enzyme (Jena Bioscience, Germany) overnight (18-20 hours) at 37 °C. Then, RFLP products were separated on a 3% agarose gel electrophoresis and visualized by Gel Doc Imaging System (E-Box VILBER, France) after staining with ethidium bromide. AluI digested the 242-bp PCR product containing the +61 A allele into 102-, 91-, 34-, and 15-bp fragments while the +61 G allele produced 193-, 34-, and 15-bp fragments. The genotyping process is shown in the Figure 1.

**Statistical analysis**

The association of EGF +61A>G (rs4444903) polymorphism with HCC risk and the Hardy-Weinberg equilibrium exact test were performed with the online SNPstats software (25). The SNPstats software analyses the associations by linear or logistic regression according to the response variable (26). This web-based software uses the multiple inheritance models (co-dominant, dominant, recessive, overdominant and log-additive) for SNP association analysis (26, 27). Based on the assumption that EGF +61A is a risk allele, these inheritance models compares A/A genotypes to G/G-A/G genotypes in recessive model and A/G-A/A to G/G genotypes in dominant model. These inheritance models, also, compare A/G genotypes to G/G-A/A genotypes in Overdominant model and A/A, A/G to G/G genotypes in Codominant model.

## Results

In the present study, the association of the EGF +61A>G (rs4444903) polymorphism with HCC was investigated in 146 Iranian subjects including 40 HCC patients and 106 healthy controls. The distribution of genotypes and alleles are shown in Table 1. Genotype frequencies of the EGF +61A>G were in Hardy Weinberg equilibrium in both groups (p>0.05). 

**Table 1:** EGF +61G/A (rs4444903) genotype and allele frequencies (n=146)

	All subjects		Control		Case	
Allele	Count	Proportion	Count	Proportion	Count	Proportion
G	149	0.51	116	0.55	33	0.41
A	143	0.49	96	0.45	47	0.59
Genotype						
A/A	35	0.24	24	0.23	11	0.28
G/A	73	0.5	48	0.45	25	0.62
G/G	38	0.26	34	0.32	4	0.1

**Table 2:** Association of EGF +61G/A (rs4444903) polymorphism with HCC (n=146, adjusted by SEX

Model	Genotype	Control (%)	Case (%)	OR (95% CI)	*P*-value
Codominant	G/G	34 (32.1%)	4 (10%)	1.00	0.012
	A/G	48 (45.3%)	25 (62.5%)	4.51 (1.43-14.19)	
	A/A	24 (22.6%)	11 (27.5%)	4.33 (1.20-15.59)	
Dominant	G/G	34 (32.1%)	4 (10%)	1.00	0.0029
	A/G-A/A	72 (67.9%)	36 (90%)	4.46 (1.46-13.63)	
Recessive	G/G-A/G	82 (77.4%)	29 (72.5%)	1.00	0.44
	A/A	24 (22.6%)	11 (27.5%)	1.41 (0.60-3.30)	
Overdominant	G/G-A/A	58 (54.7%)	15 (37.5%)	1.00	0.071
	A/G	48 (45.3%)	25 (62.5%)	1.98 (0.94-4.18)	
Log-additive	---	---	---	1.86 (1.07-3.23)	0.024
	Allele				
	G	116 (55%)	33 (41%)	1.00	
	A	96 (45%)	47(59%)	1.72 (1.02 - 2.90)	0.04

The frequency of the EGF +61A allele in HCC patients was significantly higher than the healthy controls [OR = 1.72, 95% CI (1.02 - 2.90), P value = 0.04] (Table 2). Significant association was observed between the frequency of AA and AG genotypes among HCC patients and healthy controls under co-dominant (p=0.012) and dominant (p=0.0029) models, when GG genotype was considered as a reference (Table 2). In contract, there was not significant association between the genotype frequency of HCC patients and healthy controls under recessive (p=0.44) and overdominant (p=0.071) models (Table 2).

## Discussion

Epidermal growth factor (EGF) is a mitogen for hepatocytes and plays a critical role in liver tissue regeneration (28, 29). EGF has many biological functions including induction of DNA synthesis, proliferation, differentiation, and tumorigenesis of epidermal and epithelial tissues and can activate multiple signal pathways through interaction with its receptor EGFR (30, 31). EGF +61A/G polymorphism is the most common SNP located in the 5′-untranslated region of the EGF gene (rs4444903) that influences EGF production by affecting DNA folding or gene transcription (30, 32). Association between EGF 61A>G polymorphisms and the HCC susceptibility has been reported in recent years (21, 31, 33), but the results remain inconclusive. 

In the present study, we observed that the EGF +61A/G polymorphism presented a risk factor for HCC in an Iranian population. Our data showed that the frequency of the EGF +61A allele in HCC patients was significantly higher than the healthy controls (P value = 0.04) (table 2). It seems that EGF +61A allele is risk allele for HCC patients. This finding is opposite to some studies that reported EGF +61G allele significantly associated with increased risk of HCC (34, 35). This suggests that the increase risk of HCC might be dependent on the population.

Genotypic analysis, also, showed that there are significant association between the frequency of AA and AG genotypes among HCC patients and healthy controls under co-dominant and dominant models, not under recessive and over dominant models (Table 2). The present study was performed with small sample size. However, our genetic data provide suitable information about of EGF +61A/G polymorphism and susceptibility to HCC in Iranian population, but, it is necessary to replicate the study with larger sample size. Our findings suggest that the EGF gene +61A/G polymorphism (rs4444903) might be a risk factor for susceptibility to HCC in Iranian population. *However*, further studies with large sample size should be conducted to clarifying the association of EGF gene +61A/G polymorphism (rs4444903) with HCC in Iranian population.
